# Challenges in the diagnosis of asthma in children, what are the solutions? A scoping review of 3 countries in sub Saharan Africa

**DOI:** 10.1186/s12931-022-02170-y

**Published:** 2022-09-19

**Authors:** P. Magwenzi, S. Rusakaniko, E. N. Sibanda, F. Z. Gumbo

**Affiliations:** 1 Child and Adolescent Health Unit, Faculty of Medical Sciences, University of Zimbabwe, P O Box A178, Avondale, Harare, Zimbabwe; 2 Family Medicine, Global and Public Health Unit, Faculty of Medical Sciences, University of Zimbabwe, P O Box A178, Avondale, Harare, Zimbabwe; 3 Asthma, Allergy and Immune Dysfunction Clinic, 113, Kwame Nkrumah Avenue, Harare, Zimbabwe

**Keywords:** Childhood asthma, Diagnosis, Under-diagnosis Nigeria, South Africa, Uganda, Strategies, Improving asthma diagnosis

## Abstract

**Background:**

Asthma is the commonest chronic respiratory tract disease in children. In low-income countries, challenges exist in asthma diagnosis. In surveys done in children, the prevalence of ‘asthma’ defined by symptoms is high compared to ‘doctor diagnosed asthma’. The questions answered by this review are (i) What challenges have been experienced in the diagnosis of asthma in children? (ii) What solutions will address these challenges?

**Methods:**

The Arksey and O’Malley’s framework for scoping reviews was used for the study methodology, while the PRISMA-ScR checklist guided the reporting process. Electronic databases: PubMed Central, EMBASE and Google Scholar were searched. Primary quantitative and qualitative studies and reviews from 2010 to 2021, from Nigeria, South Africa and Uganda written in English or translated to English, which answered the study questions were included. The author, title, country, study type, methods, purpose, findings and references were captured onto a predefined data collection table. The ‘Preview, Question, Read, Summarise’ system was used and a narrative report was used to summarise the findings.

**Results:**

A total of 28 studies were included. The causes of under-diagnosis of asthma include lack of community knowledge and perception of asthma, poor accessibility to health care, strained health systems, lack of diagnostic tests including spirometry, low levels of knowledge among health-care workers and lack of or non-implementation of asthma guidelines. Strategies to improve asthma diagnosis will include community and school based education programmes, revision of asthma diagnostic terms, guideline development and implementation and health systems strengthening.

**Conclusion:**

This scoping review provides research evidence for policy makers and health-workers involved in the care of asthmatic children on challenges faced in asthma diagnosis and strategies to improve asthma diagnosis.

**Supplementary Information:**

The online version contains supplementary material available at 10.1186/s12931-022-02170-y.

## Background

Asthma is the most common chronic respiratory tract disease in children. According to the Global Asthma Report of 2018, asthma was reported to affect about 339.4 million people globally and 14% of children below 15 years of age [1]. Time trends in the prevalence of reported asthma symptoms in children below 15 years of age in low to middle income countries (LMIC) have shown a proportionate increase from 12.1% in 1990 to 13.9% in 2010 [2]. The main cause of this increase is increased urbanization with the associated pollution [3]. Phase 111 ISAAC data from Africa suggest that the prevalence of childhood asthma ranges from 4 to 21.5% in children aged 13–14 years [4]. Latest data show that the highest prevalence of asthma in Sub Saharan Africa (21.3%) as measured by self-reported current wheeze has been reported in South African adolescents [5].

Children in LMIC tend to have severe asthma symptoms [6, 7]. The reason for this is not well described and may be due to lack of awareness of asthma symptoms, use of solid fuel, asthma underdiagnosis and strained health systems with inadequate asthma care [8].

No gold standard exists for asthma diagnosis [9]. A combination of the suggestive symptoms of intermittent cough, wheeze, difficulty breathing, chest tightness, family history of atopy or asthma, audible wheezes on examination and demonstration of variable airway reversibility makes the diagnosis of asthma more likely [10]. A number of international guidelines on asthma diagnosis exist including Global Initiative for Asthma (GINA) [10], National Asthma Education Prevention Programme /Expert Panel Report (NAEPP/EPR) [11] and National Institute for Care Excellence (NICE) [12].

### Evidence of the under-diagnosis and mis-diagnosis of asthma

While reviews from low income countries have revealed that there is under-diagnosis or mis-diagnosis of asthma [13–16] with a preference for infectious respiratory diseases, studies from high income countries suggest over-diagnosis of asthma. A systematic review of studies in children and adults showed that 20 to 70% of asthmatics are under-diagnosed while 30 to 35% are over-diagnosed [17]. In Sub Saharan Africa (SSA), several studies have reported that asthma is under-diagnosed in children. Prevalence of ‘asthma’ defined by symptoms was reported to be as high as 24–27% compared to the prevalence of ‘doctor diagnosed asthma’ reported to be 2.2% in community surveys in children in Nigeria [18]. Several studies done in South Africa [19–21] and Uganda [22] have revealed that childhood asthma is under-diagnosed.

## The rationale for the review

About 60–80% of asthmatic children show signs in the first 5 years of life [23, 24]. Diagnosis of asthma is crucial to precise treatment. Asthma is associated with school absence [25], repeated presentation to the emergency department [26], Chronic Obstructive Pulmonary Disease [27] and decreased lung function in adulthood [28]. Missing a diagnosis of asthma particularly in infants and children, may lead to increased morbidity and mortality due to this disease as shown in a Ugandan study [22].

To date, there has not been a review that is dedicated to report on the challenges in the diagnosis of childhood asthma and possible solutions thereof.

The main aim of this review was to summarise the evidence on diagnosis of asthma in children in Nigeria, South Africa and Uganda. This study answers these specific questions: (i) What challenges have been experienced in the diagnosis of asthma in children? (ii) What possible solutions have been suggested or implemented to curb the challenges in diagnosis of asthma in children?

## Methods

The protocol for this scoping review was registered on the Open Science Framework 10.17605/OSF.IO/RD6XT.

### Study design

The Arksey and O’Malley’s framework for scoping reviews was utilised as the methodological framework [29], while the Preferred Reporting Items for Systematic reviews and Meta-Analysis PRISMA-2020-checklist guided the process of the review and reporting of findings [30] (Additional file 1). A scoping review rather than a systematic review with meta-analysis was done because of the broad scope of the information collected and the heterogeneity of the study designs. Quantitative, qualitative studies and reviews were included in this scoping review. Inclusion of qualitative evidence was meant to capture health workers and care-givers perceptions regarding the challenges faced in asthma diagnosis and possible solutions from the viewpoint of those experiencing these challenges.

### Identification of the research questions

(i) What challenges have been experienced in the diagnosis of asthma in children?
(ii) What possible solutions have been suggested or implemented to curb the challenges in diagnosis of asthma in children?

### Identification of the relevant studies

#### Study eligibility criteria

We used the ‘Participants, Concept and Context (PCC)’ criteria to clearly define eligible studies as described in the Joanna Briggs Manual for Evidence Synthesis 2021 [31].

#### Participant

Included in this review are studies that reported on the challenges faced in the diagnosis of asthma in children from the standpoint of the researchers, children, caregivers, health-workers, and any other stakeholders involved with asthma diagnosis and management. For this review, according to the United Nations Children’s Fund (UNICEF) children are defined as persons under the age of 18 years, while healthcare workers are defined as people whose job is to protect and improve the health of their communities.

#### Concept

Studies that reported on the challenges faced in the medical diagnosis of asthma in children and/or reported, suggested, or explored strategies that maybe used to overcome these challenges were assessed for inclusion. Challenge as a noun is used synonymously with barrier, problem or setback [32] and for purposes of this review meant any factor that impedes asthma diagnosis.

#### Context

The past decade has seen major changes in the understanding of the immune-pathogenesis and diagnosis of asthma. To capture this, studies from 2010 to 2021, written in English or already found to be translated to English by their own authors or publishers, which answered the study questions were assessed for inclusion. Included are peer reviewed articles with the focus of the paper being on ‘asthma in children in Nigeria, South Africa or Uganda,’ ‘challenges in asthma diagnosis’ or ‘solutions to challenges in asthma diagnosis. Historically SSA countries have suffered from the effects of poverty, struggling economies and poor health delivery in comparison to countries to the north of the Sahara. Asthma and other non-communicable diseases have particularly been neglected with healthcare efforts biased towards infectious diseases hence the focus on countries in SSA. Prior to narrowing the scoping review to these 3 countries, a preliminary search was done on asthma in children in Sub Saharan Africa. The initial search was broad, the results showed that research on childhood asthma is dominated by authors from mainly three countries: Nigeria, South Africa and Uganda. In addition, it became clearer that a closer review and analysis of data published from these 3 countries, would allow a more focused view of the relevant local factors applicable to the individual countries.

#### Study exclusion criteria

Studies that were not available in English, books, conference abstracts, expert papers, editorials and grey literature were excluded from this review.

#### Study type

Primary quantitative and qualitative studies and reviews that met the eligibility criteria were included.

#### Search strategy

For each of the two objectives, electronic databases PubMed, Embase, MEDLINE and Scopus were searched. A systematic search strategy was developed with the help of the college librarian (MM) using a combination of Medical Subject Headings (MeSH terms) and controlled vocabulary to identify peer reviewed articles answering the research objectives.

#### Objective 1 (Search 1) Challenges in the diagnosis of asthma in children

MeSH terms: ‘asthma’, ‘diagnosis’, ‘underdiagnosis’, ‘children’, ‘paediatric’, ‘Nigeria’, ‘South Africa’, ‘Uganda’, using AND/OR in the search builder.

#### Objective 2 (Search 2) Solutions to challenges in asthma diagnosis in children

MeSH terms: ‘improving asthma diagnosis’, ‘solutions to challenges in asthma diagnosis’ ‘underdiagnosis’ ‘children’, ‘paediatric’, ‘Nigeria’, ‘South Africa’, ‘Uganda’ ‘challenges’, using AND/OR in the search builder.

These MeSH terms generated under the two objectives were used to develop two separate search strings which were used to search PubMed, Embase, MEDLINE and Scopus.

Table 1 below summarises the eligibility criteria.

**Table 1:** Eligibility criteria

Research objective	To report on the challenges in diagnosis of asthma in children	To report on the solutions to challenges faced in diagnosis of asthma in children
Population/participants	Asthma diagnosis in children from the standpoint of the researchers, children, caregivers, health-workers, and any other stakeholders involved with asthma diagnosis and management	Solutions to improve diagnosis of asthma in children from the standpoint of the researchers, children, caregivers, health-workers, and any other stakeholders involved with asthma diagnosis and management
Concept	Challenges/problems/barriers to the medical diagnosis of asthma in children	Solutions or strategies that have been suggested or used to overcome challenges in asthma diagnosis in children
Context	Nigeria, South Africa, Uganda, 2010 to 2021	Nigeria, South Africa, Uganda, 2010 to 2021
Study type	Primary quantitative and qualitative studies, reviews	Primary quantitative and qualitative studies, reviews
Exclusion criteria	Studies not available in English, books, conference abstracts editorials, expert/opinion papers	Studies not available in English, books, conference abstracts editorials, expert/opinion papers

### Study selection process

Two reviewers PM and FZG conducted the database search. They screened the results against the eligibility criteria. The studies selected for inclusion by title then had their abstracts retrieved and assessed against the eligibility criteria, for eligible abstracts, the full articles were analysed against the eligibility criteria.

#### Data extraction and charting onto a predefined data charting form

Relevant data was extracted and charted from the included studies. The Preview, Question, Read and Summarize (PQRS) system was used on all the studies included. From each of the included studies the author, country, study type, findings and full reference, were captured onto a pre-defined data extraction chart (Additional file 2). The studies that met the inclusion criteria were grouped under the subheadings: “challenges in asthma diagnosis” and “solutions to improve asthma diagnosis”. Two independent members of the review team (ENS, SR) assessed the studies that were included. Disagreements among reviewers on whether an article was suitable for inclusion were resolved by these two independent members of the review team (ENS, SR).

### Collating, summarising, and reporting the results into tables and charts

The key findings were collated, summarised and reported in tables and charts. For the studies included for the review; relationships between studies were explored. The PRISMA flow diagram was used to summarise the number of records identified, included and excluded and the reasons for exclusion. (Additional file 3).

#### Data analysis

For quantitative data descriptive statistics were used. The findings were sorted into broad themes. This enabled identification, analysis and interpretation the findings from the articles according to key themes or patterns. For the one study with qualitative data, content analysis was done.

#### Data presentation

A narrative report was used to summarise the findings to meet the objectives of the scoping review.

## Results

### Study characteristics

The initial search using the search strategy for Objective 1: Challenges in the diagnosis of asthma in children (Search 1) gave a total of 168 while Objective 2/Solutions to challenges in the diagnosis of asthma in children (Search 2) yielded a total of 122. After applying the eligibility criteria to titles and abstracts, 59 full articles for Objective 1 and 34 full articles for Objectives 2 were retrieved and analysed. Of these 25 articles meeting the eligibility criteria were retained. References in each of the identified papers were screened and 3 additional articles met the eligibility criteria to make a total of 28. The PRISMA Flow Chart (Additional file 3) illustrates the study selection process.

Most of the studies included utilized quantitative methods mostly cross-sectional.

Table 2 summarizes the study characteristics of the included studies.

**Table 2:** Retained study by country N = 28

Country	Challenges in asthma diagnosis (citation) n = 19	Solutions to challenges in asthma diagnosis (citation) n = 16
Nigeria n = 17	[8, 18, 33, 35–45]	[8, 35, 38, 42, 43, 47, 48, 54]
South Africa n = 6	[19, 20]	[20, 50, 51, 53, 55]
Uganda n = 5	[22, 34, 46]	[22, 49, 52]

For both objectives, the included studies utilized mostly quantitative methods, only one study utilized qualitative methods. Additional file 3 summarizes the study distribution by author, country, study type and findings of the retained studies.

### Challenges in the diagnosis of asthma in children

**Fig. 1:**
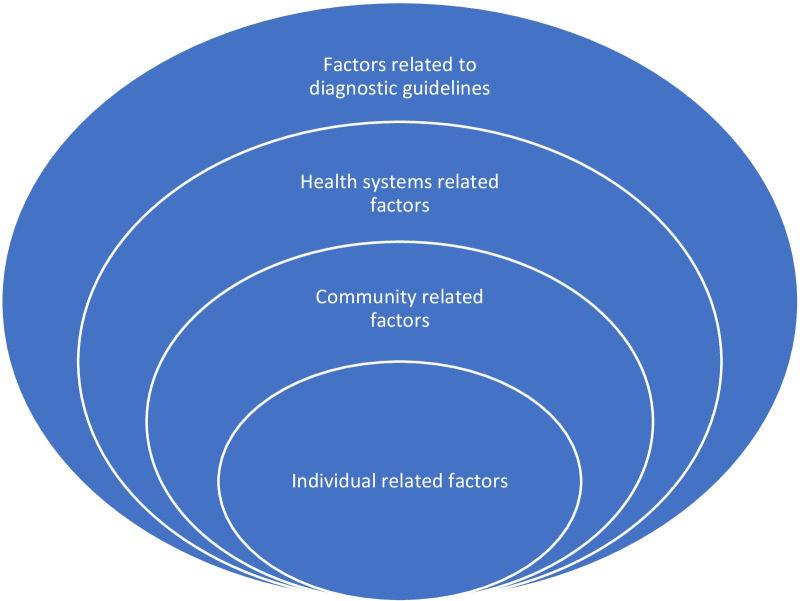
Factors leading to underdiagnosis of asthma

Figure 1 depicts the reasons for the under-diagnosis of asthma in children that have been put forward by various authors.

#### (i) Individual related factors

##### Lack of awareness of asthma symptoms

Lack of awareness of symptoms of asthma by the children and caregivers has been cited as one of the reasons for possible underdiagnosis of asthma [8, 20]. In Nigeria there were misconceptions and low levels of knowledge about asthma among parents of asthmatic children. Only 27.5% of the caregivers of asthmatic children knew that cough, wheeze and shortness of breath were the main symptoms of asthma [33].

#### (ii) Community related factors

##### Lack of asthma related terminology in local languages

Two reviews done on South African studies, showed that asthma related terminology including ‘asthma’ and ‘wheeze’ are absent in some local, ethnic languages [19, 20].

##### Stigma associated with asthma

A qualitative study in Uganda by van Gemert et al. revealed that while respiratory symptoms were common in children as well as adults, there was stigma associated with the presence of respiratory symptoms leading to caregivers not reporting symptoms [34]. In addition, the presence of respiratory symptoms was associated with tuberculosis hence act as an impediment to asthma diagnosis.

#### (iii) Health systems related factors

##### Poverty and inaccessibility to health care

In several studies, poor socio-economic status and/or lack of medical health funding was associated with under-recognition of asthma hence severe asthma [8, 19].

Inaccessibility to health facilities has been cited by Oluwole et al. as one of the reasons for possible underdiagnosis of asthma [18]. A review of studies done in South Africa on childhood asthma by Zar et al. highlighted inaccessibility to health care as an impediment to asthma diagnosis [19].

##### Strained health systems and lack of diagnostic resources

Health systems with strained resources have been cited by some authors as the cause for under-diagnosis of asthma [19].

While lung function testing eg spirometry remains an objective measure in diagnosis and assessing the severity of airflow obstruction, it remains widely unavailable [18, 35–37]. Spirometry, skin prick allergy testing and serum specific immunoglobulin E (IgE) for allergy testing are not available in most primary care, district and provincial hospitals where the asthmatic patient initially presents. The availability of spirometry was reported to be as low as 29.4% in Nigeria while availability of the less reliable peak flow meter which may be used in diagnosis and monitoring was reported to be 38% [38]. A study done by Desalu et al. in Nigeria showed that while 60.7% knew of the role of spirometry in diagnosis, only 25.2% reported having a spirometer in their hospital [39]. Another study in Uganda also revealed non availability of diagnostic tests [40].

Ayuk et al. reviewed literature on the use of spirometry particularly in resource poor countries and highlighted that unavailability of spirometers, ignorance of their importance in diagnosis and follow-up as well as lack of knowledge on how to use them and interpret results are the main reasons why spirometer use is low [35].

A systematic review done by Kibirige et al. on ‘Availability and Affordability of diagnostic tests for asthma and COPD’, availability of spirometry was low ranging from 13 to 53% in most countries in Sub Saharan Africa [41].

A review of records over two and half years at University of Nigeria teaching hospital spirometry laboratory showed that referral of patients from primary care health workers is poor and only patients above 15 years of age were documented to have been referred for spirometry [42]. In addition there is lack of respiratory physicians in most tertiary hospitals [43].

##### Low levels of knowledge on asthma diagnosis amongst health care workers

There is low levels of knowledge among health personnel on the utility of spirometry in the diagnostic evaluation of asthmatic children [35, 36]. In a study done in Nigeria, it was shown that while up to 95% of paediatric registrars knew and had seen a spirometer before and knew about its role in asthma diagnosis only 37% used it in diagnosis [37]. A survey among 131 health workers in 6 centres in Nigeria revealed that 116 (88.5%) had low knowledge levels on asthma diagnosis [44].

#### (iv) Factors related to diagnostic guidelines

##### Lack of or non-adherence to guidelines for asthma diagnosis

Most countries in SSA either do not have guidelines and in countries where they are available, they are not fully implemented especially in rural areas [19, 37]. Ayuk et al. showed that the use of guidelines is poor amongst paediatric residents from 23 university teaching hospitals in Nigeria with only 59.1% of them adhering to GINA guidelines [45]. The most reported reason for non-adherence to guideline based asthma diagnosis was the lack of knowledge of contents of the guideline and the importance of their role in diagnosis.

##### Mis-diagnosis of asthma due to inherent asthma heterogeneity, co-morbid conditions and alternate diagnosis

In LMIC, where the burden of infectious diseases over-ride non-communicable diseases, health workers tend to prioritize, even over-diagnose these infectious diseases at the expense of asthma. Two such conditions that tend to be over-diagnosed at the expense of asthma are bronchiolitis and pneumonia [46]. Pneumonia which mimics asthma especially if fever is not considered has been shown to lead to underdiagnosis of asthma in Ugandan children [22]. The different phenotypic presentations of the wheezing child below 5 years has led to diagnostic terms such as ‘the asthma syndrome’ being used in the Ugandan study.

### Solutions to the challenges in diagnosis of asthma in children

**Fig. 2:**
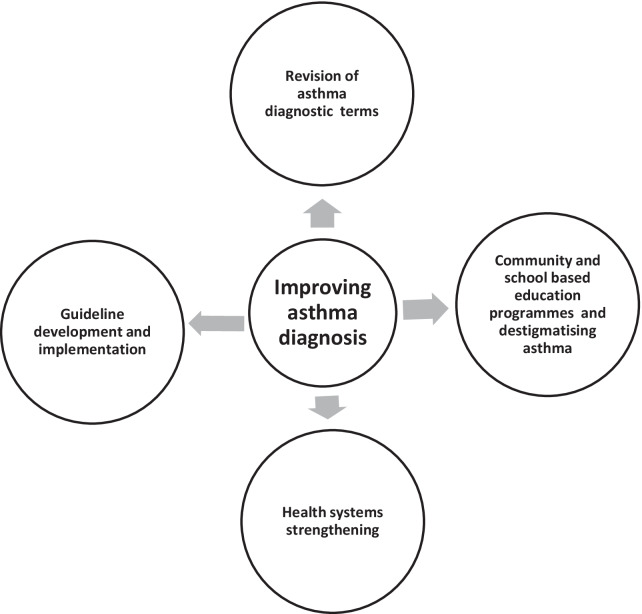
The four prongs of improving asthma diagnosis

Figure 2 summarises solutions to challenges in the diagnosis of asthma in children after synthesis of the evidence from literature.

Several authors have recommended strategies to improve asthma diagnosis based on evidence. Table 3 summarises evidence for solutions to challenges in the diagnosis of asthma in children.

**Table 3:** Solutions to the challenges in the diagnosis of asthma in children

Solution	Explanation	Sources
1. Community education and destigmatising asthma		
1a. Community education	Recommended school-based curriculum and peer driven education programmes to increase perception and recognition of asthma symptoms	Temitayo et al. [47]
1b. Community and school-based screening programmes for childhood asthma	Set up community and school-based screening programmes for childhood asthma and referral to care	Oluwole et al. [8], Kuti et al. [48]
2. Asthma diagnostic terms		
2a. Asthma diagnostic terms	Diagnostic terms like ‘wheezing disorder, the asthma syndrome, episodic viral wheeze, multiple-trigger wheeze’ for children below the age of 5 years allows for trial medication to be given until objective diagnostic re-evaluation using spirometry at 5 years of age	Nantanda et al. [22], Masekela et al. [20], Ostergaard et al. [49]
2b. Redefinition of WHO IMCI algorithms for pneumonia to include fever	Need for revision of WHO IMCI guidelines to include ‘fever’ to ‘cough’ and ‘fast breathing’ to differentiate pneumonia from asthmatics who may not have fever	Nantanda et al. [22]
2c. Asthma should be considered a strong differential diagnosis for pneumonia	Asthmatic children likely to present several times with cough wheeze and shortness of breath	Nantanda et al. [22]
3. Guideline development and implementation		
3a. Guideline development and implementation	Guidelines are effective in improving asthma diagnosis, management and outcomes in primary health-care (PHC) clinics	du Plessis et al. [50]
3b. Evidence based guidelines	Need to exclude all other causes of wheeze, therapeutic trial of inhaled steroids may be useful where objective spirometry is not available Based on evidence, a four step diagnostic process was developed	Masekela et al. on behalf of South African Child Asthma Working Group [20], van Niekerk et al. [51]
3c. Symptom-based asthma diagnosis	Simple symptom-based questionnaires have been found to be useful in asthma diagnosis in children under 5 years	Nantanda et al. [52]
4. Health systems strengthening		
4. Health systems strengthening	Investigated the use of the Practical Approach to Care Kit for Children (PACK) kit which comprise a clinical decision support tool, diagnostic algorithms, training programme and health system strengthening with health-worker supervision, regular updates and policy change. Based on this investigation, a strategy to optimise the use of PACK was proposed and may act as the basis of improving asthma diagnosis and care in addition to other acute and chronic childhood illnesses	Murdoch et al. [53]
a. Accessibility to health care	Primary health care (PHC) is key in improving asthma diagnosis in resource poor settings	du Plessis et al. [50]
b. Health worker education	Training health workers at all levels of the health system	Murdoch et al. [53]
c. Capacitation of health facilities with diagnostic equipment and asthma drugs	Need for innovative confirmatory tests for childhood asthma for children under 5 years of age	Nantanda et al. [22]
	Availing spirometry to confirm asthma diagnosis. Training health workers on use of spirometry	Ayuk et al. [35], Desalu et al. [38], Nwosu et al. [42], Obaseki et al. [43], Adeyeye et al. [54], Masekela et al. [55]

## Discussion

Globally, the shift in asthma care is towards precision medicine, with phenotyping and endotyping of asthmatic patients guiding patient tailored biologic therapy. However, SSA is lagging behind these innovations. SSA is known to have suffered the effects of poverty, struggling economies and poor health delivery systems. Despite the rising burden of childhood asthma in SSA and the associated morbidity and mortality, research on asthma in SSA is dominated by South Africa, Nigeria and Uganda. By reviewing data from these three countries, this review has summarised the challenges faced in diagnosis of asthma and the solutions thereof.

### Challenges in asthma diagnosis

A review of asthma in developing countries revealed some challenges similar to those reported in this review [56]. Poverty and inaccessibility to healthcare have been reported as factors leading to the underdiagnosis of asthma [57]. Lack of appropriate terms for asthma in local languages have been described as a deterrent to asthma diagnosis and care in an earlier review of Asthma in Africa done by Wjst [13]. Most of the studies that reported of lack of knowledge on asthma and poor perception of symptoms by both patients and caregivers suggested community based awareness programmes targeting schools [58].

Unavailability of diagnostic tests and poor knowledge levels on asthma diagnosis has been cited in adult studies a major factor in asthma underdiagnosis [59, 60]. A qualitative study in rural Asia reported that of the 22 primary healthcare professional interviewed, none had made a diagnosis of asthma in children less than 5 years of age preferring instead infectious causes [61].

### Solutions to challenges in diagnosis of childhood asthma

Communities need to adopt the worldwide charter for all children with asthma which summarise how communities and government should improve asthma diagnosis and care [62]. Because some local languages lack the terminology for wheeze, video based questionnaires may improve the sensitivity of screening for asthma [4]. Targeted education programmes on the symptoms, diagnosis, and management of asthma improves the level of knowledge among communities. This proved to be effective in a study done by Rastogi et al. evaluating the effect of educational programmes among 268 Hispanic and African American primary caregivers of asthmatic children that had repeated emergency department visits [63]. Similar improvement in knowledge levels was found among caregivers in India [64]. Symptom-based and spirometry based screening programmes have been tested in school aged children in high income countries and found to be feasible [65].

Ait Khaled et al. and Martins et al. in systematic reviews highlighted several areas meant to improve asthma care [4, 60]. These were equipment capacitation of hospitals, implementation of guidelines, policies aimed at reduction of tobacco use and provision of generic inhaled corticosteroids. The asthmatic child is most likely to present first to a primary health centre therefore strengthening and equipping these facilities to enhance asthma diagnosis and management is important for successful outcomes. The primary health care model for non-communicable disease was validated in the 2 year prospective interventional studies in South Africa [66]. In addition nurse-led primary health care for non-communicable diseases has proved to be effective in improving asthma diagnosis and care in Cameroon [67]. Recommendations by the South African Childhood Asthma Working Group (SACAWG) suggested that training health workers will improve diagnosis of childhood asthma [68].

Given that 60–80% of asthmatics present within the first 5 years of life [23] and spirometry is widely unavailable in LMIC, symptom based diagnosis becomes a reasonable option provided alternative diagnoses are looked for and excluded. In addition, efforts should be made to avail spirometry or peak flow meter in all facilities caring for children.

### Study limitations

While the authors initially set out to review studies in SSA, it became clear that there is paucity of research on asthma in children in most SSA countries, with most studies being from Nigeria, South Africa and Uganda. It is in this regard therefore that while this scoping review has come up with useful recommendations to improve asthma diagnosis in children, it should be appreciated that these findings from these 3 countries in SSA.

## Conclusions

Asthma is a major cause of child morbidity and mortality and its prevalence is rising in SSA. Early asthma diagnosis and follow-up care allow alveoli and airway health, improve lung function, and prevent the risk of COAD. Asthma diagnosis remains a challenge in SSA countries mainly due to community stigma and lack of knowledge, inaccessibility to health care, strained health systems and lack of guidelines or non-implementation of these guidelines. A comprehensive 4-pronged approach to improve asthma diagnosis will include community and school based education programmes on asthma, revision of asthma diagnostic terms, development and implementation of diagnostic guidelines and health systems strengthening. In addition there is need to improve availability and accessibility to asthma treatment.

## Supplementary Information

**Additional file 1.** PRISMA 2020 checklist.

**Additional file 2.** Data extraction form.

**Additional file 3.** PRISMA 2020 flow diagram for new systematic reviews which included searches of databases and registers only.

## Data Availability

The raw data obtained from this scoping review is available from the authors on request.

## Abbreviations

COAD: Chronic obstructive airways disease
EIB: Exercise induced bronchospasm
NAEPP/EPR: National Asthma Education Prevention Programme/Expert Panel
NICE: National Institute for Care Excellence
GINA: Global Initiative for Asthma
HIV: Human immune deficiency virus
ISAAC: The International Study of Asthma and Allergies in Childhood
JBI: Joanna Briggs Institute’s
LMIC: Low- and Middle-Income Countries
PRISMA: Preferred Reporting Items for Systematic reviews and Meta-Analyses
PQRS: Preview, Question, Read, Summarize
SSA: Sub Saharan Africa
TB: Tuberculosis
UNICEF: United Nations Children’s Fund
