# Supplementary material for: Challenges in the diagnosis of asthma in children, what are the solutions? A scoping review of 3 countries in sub Saharan Africa
Source: Respir Res. 2022 Sep 19;23:254. doi: 10.1186/s12931-022-02170-y (PMC9487077; doi:10.1186/s12931-022-02170-y)
Supplement: Supplementary file 1 — Additional file 1. PRISMA 2020 checklist. [file 12931_2022_2170_MOESM1_ESM.docx]

| **Section and Topic** | **Item #** | **Checklist item** | **Location where item is reported** |
| --- | --- | --- | --- |
| **TITLE** | | |  |
| Title | 1 | *Challenges in the diagnosis of asthma in children, what are the solutions? A 2010 to 2021 Scoping review of Nigeria, South Africa and Uganda.* | Title page/page 1 |
| **ABSTRACT** | | |  |
| Abstract | 2 | ***Background***  *Asthma is the commonest chronic respiratory tract disease in children. In low-income countries, challenges exist in asthma diagnosis. In surveys done in children, the prevalence of ‘asthma’ defined by symptoms is high compared to ‘doctor diagnosed asthma’. The questions answered by this review are (i)What challenges have been experienced in the diagnosis of asthma in children? (ii)What solutions will address these challenges?*  ***Methods***  *The Arksey and O’Malley’s framework for scoping reviews was used for the study methodology, while the PRISMA-ScR checklist guided the reporting process. Electronic databases:* *PubMed Central, EMBASE and Google Scholar were searched. Primary quantitative and qualitative studies and reviews from 2010 to 2021, from Nigeria, South Africa and Uganda written in English or translated to English, which answered the study questions were included. The author, title, country, study type, methods, purpose, findings and references were captured onto a predefined data collection table. The ‘Preview, Question, Read, Summarise' system was used and a narrative report was used to summarise the findings.*  ***Results***  *A total of 28 studies were included. The causes of under-diagnosis of asthma include lack of community knowledge and perception of asthma, poor accessibility to health care, strained health systems, lack of diagnostic tests including spirometry, low levels of knowledge among health-care workers and lack of or non-implementation of asthma guidelines. Strategies to improve asthma diagnosis will include community and school based education programmes, revision of asthma diagnostic terms, guideline development and implementation and health systems strengthening.*  ***Conclusion*** *This scoping review provides research evidence for policy makers and health-workers involved in the care of asthmatic children on challenges faced in asthma diagnosis and strategies to improve asthma diagnosis.*  ***Keywords*** *Childhood asthma, diagnosis, under-diagnosis Nigeria, South Africa, Uganda, barriers, strategies, improving asthma diagnosis* | Page 2 |
| **INTRODUCTION** | | |  |
| Rationale | 3 | About 60-80% of asthmatic children show signs in the first 5 years of life (23) (24). Diagnosis of asthma is crucial to precise treatment. Asthma is associated with school absence (25), repeated presentation to the emergency department (26), Chronic Obstructive Pulmonary Disease (27) and decreased lung function in adulthood (28). Missing a diagnosis of asthma particularly in infants and children, may lead to increased morbidity and mortality due to this disease as shown in a Ugandan study (22).  To date, there has not been a review that has reported on the challenges in the diagnosis of childhood asthma and possible solutions thereof.  The main aim of this review was to summarise the evidence on diagnosis of asthma in children in Nigeria, South Africa and Uganda. | Page 4 |
| Objectives | 4 | (i)To report on challenges experienced in the diagnosis of asthma in children.  (ii) To report on the solutions that have been recommended or implemented to curb the challenges in diagnosis of asthma in children? | Page 4 |
| **METHODS** | | |  |
| Eligibility criteria | 5 | **Table 1: Eligibility criteria**   \| **Research Objective** \| **To report on the challenges in diagnosis of asthma in children** \| **To report on the solutions to challenges faced in diagnosis of asthma in children** \| \| --- \| --- \| --- \| \| **Population/Participants** \| Asthma diagnosis in children from the standpoint of the researchers, children, caregivers, health-workers, and any other stakeholders involved with asthma diagnosis and management \| Solutions to improve diagnosis of asthma in children from the standpoint of the researchers, children, caregivers, health-workers, and any other stakeholders involved with asthma diagnosis and management \| \| **Concept** \| Challenges/problems/barriers to the medical diagnosis of asthma in children \| Solutions or strategies that have been suggested or used to overcome challenges in asthma diagnosis in children \| \| **Context** \| Nigeria, South Africa, Uganda, 2010 to 2021 \| Nigeria, South Africa, Uganda, 2010 to 2021 \| \| **Study Type** \| Primary quantitative and qualitative studies, reviews \| Primary quantitative and qualitative studies, reviews \| \| **Exclusion criteria** \| Studies not available in English, books, conference abstracts editorials, expert/opinion papers. \| Studies not available in English, books, conference abstracts editorials, expert/opinion papers. \| | Page 7 |
| Information sources | 6 | For each of the two objectives, electronic databases PubMed, Embase, MEDLINE and Scopus were searched. Reference lists of included studies were also perused for relevant studies. | Page 6 |
| Search strategy | 7 | **Search strategy**  For each of the two objectives, electronic databases PubMed, Embase, MEDLINE and Scopus were searched. A systematic search strategy was developed with the help of the college librarian (MM) using a combination of Medical Subject Headings (MeSH terms) and controlled vocabulary to identify peer reviewed articles answering the research objectives  **Objective 1 (Search 1) Challenges in the diagnosis of asthma in children**  MeSH terms: ‘asthma’, ‘diagnosis’, ‘underdiagnosis’, ‘children’, ‘paediatric’, ‘Nigeria’, ‘South Africa’, ‘Uganda’, using AND/OR in the search builder.  **Objective 2 (Search 2) Solutions to challenges in asthma diagnosis in children**  MeSH terms: ‘improving asthma diagnosis’, ‘solutions to challenges in asthma diagnosis’ ‘underdiagnosis’ ‘children’, ‘paediatric’, ‘Nigeria’, ‘South Africa’, ‘Uganda’ ‘challenges’, using AND/OR in the search builder.  These MeSH terms generated under the two objectives were used to develop two separate search strings which were used to search PubMed, Embase, MEDLINE and Scopus | Page 6 |
| Selection process | 8 | Two reviewers PM and FZG conducted the database search. They screened the results against the eligibility criteria. The studies selected for inclusion by title then had their abstracts retrieved and assessed against the eligibility criteria, for eligible abstracts, the full articles were analysed against the eligibility criteria. | Page 7 |
| Data collection process | 9 | Relevant data was extracted and charted from the included studies. The Preview, Question, Read and Summarize (PQRS) system was used on all the studies included. From each of the included studies the author, country, study type, findings and full reference, were captured onto a pre-defined data extraction chart. (Supplement 2). The studies that met the inclusion criteria were grouped under the subheadings: “challenges in asthma diagnosis” and “solutions to improve asthma diagnosis”. Two independent members of the review team (ENS, SR) assessed the studies that were included. Disagreements among reviewers on whether an article was suitable for inclusion were resolved by these two independent members of the review team (ENS, SR). | Page 8 |
| Data items | 10a | From each of the included studies the following: author, country, study type, findings and full reference, were captured onto a pre-defined data extraction chart. | Supplement 2 |
|  | 10b | Not applicable. |  |
| Study risk of bias assessment | 11 | Methodological quality appraisal and risk of bias assessment for the included studies was not done, the authors felt this scoping review aim was to map the evidence around asthma diagnosis particularly as it relates to the challenges faced and solutions to improve asthma diagnosis as reported in literature rather than compare diagnostic criteria. In that regard as a ‘mapping review’ even without quality appraisal may be adequate to give guidance on how asthma diagnosis may be improved. |  |
| Effect measures | 12 | Not applicable |  |
| Synthesis methods | 13a | For quantitative data descriptive statistics were used. The findings were sorted into broad themes. This enabled identification, analysis and interpretation the findings from the articles according to key themes or patterns. For the one study with qualitative data, content analysis was done. | Page 9 |
|  | 13b | Describe any methods required to prepare the data for presentation or synthesis, such as handling of missing summary statistics, or data conversion: Not applicable |  |
|  | 13c | **Describe any methods used to tabulate or visually display results of individual studies and syntheses.** Relevant data was extracted and charted from the included studies. The Preview, Question, Read and Summarize (PQRS) system was used on all the studies included. From each of the included studies the author, country, study type, findings and full reference, were captured onto a pre-defined data extraction chart. (Supplement 3). The studies that met the inclusion criteria were grouped under the subheadings: “challenges in asthma diagnosis” and “solutions to improve asthma diagnosis”. The key findings were collated, summarised and reported in tables and charts. For the studies included for the review; relationships between studies were explored. |  |
|  | 13d | **Describe any methods used to synthesize results and provide a rationale for the choice(s).** If meta-analysis was performed, describe the model(s), method(s) to identify the presence and extent of statistical heterogeneity, and software package(s) used : Not applicable |  |
|  | 13e | Describe any methods used to explore possible causes of heterogeneity among study results (e.g. subgroup analysis, meta-regression). Not applicable |  |
|  | 13f | Describe any sensitivity analyses conducted to assess robustness of the synthesized results. Not applicable |  |
| Reporting bias assessment | 14 | Risk of bias assessment for the included studies was not done, the authors felt this scoping review aim was to map the evidence around asthma diagnosis particularly as it relates to the challenges faced and solutions to improve asthma diagnosis as reported in literature rather than compare diagnostic criteria. In that regard as a ‘mapping review’ even without quality appraisal may be adequate to give guidance on how asthma diagnosis may be improved. |  |
| Certainty assessment | 15 | Describe any methods used to assess certainty (or confidence) in the body of evidence for an outcome. Not applicable |  |
| **RESULTS** | | |  |
| Study selection | 16a | Describe the results of the search and selection process, from the number of records identified in the search to the number of studies included in the review, ideally using a flow diagram. | Supplement 3 |
|  | 16b | Cite studies that might appear to meet the inclusion criteria, but which were excluded, and explain why they were excluded. | Supplement 3 |
| Study characteristics | 17 | Cite each included study and present its characteristics. Table 2, Table 3, Supplement 2: Data extraction Chart. | Table 2, Table 3, Supplement 2 |
| Risk of bias in studies | 18 | Present assessments of risk of bias for each included study. Not applicable |  |
| Results of individual studies | 19 | For all outcomes, present, for each study: (a) summary statistics for each group (where appropriate) and (b) an effect estimate and its precision (e.g. confidence/credible interval), ideally using structured tables or plots. |  |
| Results of syntheses | 20a | For each synthesis, briefly summarise the characteristics and risk of bias among contributing studies. Not applicable |  |
|  | 20b | Present results of all statistical syntheses conducted. If meta-analysis was done, present for each the summary estimate and its precision (e.g. confidence/credible interval) and measures of statistical heterogeneity. If comparing groups, describe the direction of the effect. Not applicable |  |
|  | 20c | Present results of all investigations of possible causes of heterogeneity among study results. Not applicable |  |
|  | 20d | Present results of all sensitivity analyses conducted to assess the robustness of the synthesized results. Not applicable |  |
| Reporting biases | 21 | Present assessments of risk of bias due to missing results (arising from reporting biases) for each synthesis assessed. Not applicable |  |
| Certainty of evidence | 22 | Present assessments of certainty (or confidence) in the body of evidence for each outcome assessed. Not applicable |  |
| **DISCUSSION** | | |  |
| Discussion | 23a | **Provide a general interpretation of the results in the context of other evidence.**  Globally, the shift in asthma care is towards precision medicine, with phenotyping and endotyping of asthmatic patients guiding patient tailored biologic therapy. However, SSA is lagging behind these innovations. SSA is known to have suffered the effects of poverty, struggling economies and poor health delivery systems. Despite the rising burden of childhood asthma in SSA and the associated morbidity and mortality, research on asthma in SSA is dominated by South Africa, Nigeria and Uganda. By reviewing data from these three countries, this review has summarised the challenges faced in diagnosis of asthma and the solutions thereof.  **Challenges in asthma diagnosis**  A review of asthma in developing countries revealed some challenges similar to those reported in this review (56). Poverty and inaccessibility to healthcare have been reported as factors leading to the underdiagnosis of asthma (57). Lack of appropriate terms for asthma in local languages have been described as a deterrent to asthma diagnosis and care in an earlier review of Asthma in Africa done by Wjst (13). Most of the studies that reported of lack of knowledge on asthma and poor perception of symptoms by both patients and caregivers suggested community based awareness programmes targeting schools (58).  Unavailability of diagnostic tests and poor knowledge levels on asthma diagnosis has been cited in adult studies a major factor in asthma underdiagnosis (59), (60). A qualitative study in rural Asia reported that of the 22 primary healthcare professional interviewed, none had made a diagnosis of asthma in children less than 5 years of age preferring instead infectious causes (61).  **Solutions to challenges in diagnosis of childhood asthma**  Communities need to adopt the worldwide charter for all children with asthma which summarise how communities and government should improve asthma diagnosis and care (62). Because some local languages lack the terminology for wheeze, video based questionnaires may improve the sensitivity of screening for asthma (4). Targeted education programmes on the symptoms, diagnosis, and management of asthma improves the level of knowledge among communities. This proved to be effective in a study done by Rastogi et al evaluating the effect of educational programmes among 268 Hispanic and African American primary caregivers of asthmatic children that had repeated emergency department visits (63). Similar improvement in knowledge levels was found among caregivers in India (64). Symptom-based and spirometry based screening programmes have been tested in school aged children in high income countries and found to be feasible. (65).  Ait Khaled et al and Martins et al in systematic reviews highlighted several areas meant to improve asthma care (4), (60). These were equipment capacitation of hospitals, implementation of guidelines, policies aimed at reduction of tobacco use and provision of generic inhaled corticosteroids. The asthmatic child is most likely to present first to a primary health centre therefore strengthening and equipping these facilities to enhance asthma diagnosis and management is important for successful outcomes. The primary health care model for non-communicable disease was validated in the 2 year prospective interventional studies in South Africa (66). In addition nurse-led primary health care for non-communicable diseases has proved to be effective in improving asthma diagnosis and care in Cameroon (67). Recommendations by the South African Childhood Asthma Working Group (SACAWG) suggested that training health workers will improve diagnosis of childhood asthma (68).  Given that 60-80 % of asthmatics present within the first 5 years of life (23) and spirometry is widely unavailable in LMIC, symptom based diagnosis becomes a reasonable option provided alternative diagnoses are looked for and excluded. In addition, efforts should be made to avail spirometry or peak flow meter in all facilities caring for children. | Page 14 |
|  | 23b | **Recommendations**  A comprehensive 4-pronged approach to improve asthma diagnosis will include community and school based education programmes on asthma, revision of asthma diagnostic terms, development and implementation of diagnostic guidelines and health systems strengthening. In addition there is need to improve availability and accessibility to asthma treatment. |  |
|  | 23c | **Discuss any limitations of the review processes used.**  This study is limited by being a scoping review. While a scoping review is superior in the level of evidence than a general literature review, it lags behind a systematic review with meta-analysis. But the broad and heterogeneous nature of the information gathered to answer the review questions prevented us from using the Cochrane or Joanna Briggs methodology. In this review, studies with a wide range of methodologies were included ie. community based, cross-sectional studies, case control and cohort studies. While the authors initially set out to review studies in SSA, it became clear that there is paucity of research on asthma in children in most SSA countries, with most studies being from Nigeria, South Africa and Uganda. It is in this regard therefore that while this scoping review has come up with useful recommendations to improve asthma diagnosis in children, it should be appreciated that these findings are from these 3 countries in SSA. | Page 17 |
|  | 23d | **Discuss implications of the results for practice, policy, and future research.**  Asthma diagnosis remains a challenge in SSA countries mainly due to community stigma and lack of knowledge , inaccessibility to health care, strained health systems and lack of guidelines or non-implementation of these guidelines. A comprehensive 4-pronged approach to improve asthma diagnosis will include community and school based education programmes on asthma, revision of asthma diagnostic terms, development and implementation of diagnostic guidelines and health systems strengthening. In addition there is need to improve availability and accessibility to asthma treatment. | Page 17 |
| **OTHER INFORMATION** | | |  |
| Registration and protocol | 24a | The protocol for this scoping review was registered on the Open Science Framework 10.17605/OSF.IO/RD6XT. |  |
|  | 24b | Indicate where the review protocol can be accessed, or state that a protocol was not prepared. The protocol for this scoping review was registered on the Open Science Framework 10.17605/OSF.IO/RD6XT. |  |
|  | 24c | Describe and explain any amendments to information provided at registration or in the protocol. Made amendments and corrections to the methodology as guided by the review team. Narrowed the search to 3 countries in SSA Nigeria, South Africa and Uganda . |  |
| Support | 25 | This study was funded by TIBA which is also supporting PM for her fieldwork on ‘Towards improving asthma diagnosis in children.’ PM is a TIBA fellow. |  |
| Competing interests | 26 | The authors declare that they do not have competing interests. |  |
| Availability of data, code and other materials | 27 | The raw data obtained from this scoping review is available from the authors on request. |  |

*From:*  Page MJ, McKenzie JE, Bossuyt PM, Boutron I, Hoffmann TC, Mulrow CD, et al. The PRISMA 2020 statement: an updated guideline for reporting systematic reviews. BMJ 2021;372:n71. doi: 10.1136/bmj.n71

For more information, visit: <http://www.prisma-statement.org/>
