# Supplementary material for: Challenges in the diagnosis of asthma in children, what are the solutions? A scoping review of 3 countries in sub Saharan Africa
Source: Respir Res. 2022 Sep 19;23:254. doi: 10.1186/s12931-022-02170-y (PMC9487077; doi:10.1186/s12931-022-02170-y)
Supplement: Supplementary file 2 — Additional file 2. Data extraction form. [file 12931_2022_2170_MOESM2_ESM.docx]

Additional file 2: Data extraction Form

| Objective 1: Challenges in asthma diagnosis (N=19) | | | |
| --- | --- | --- | --- |
| (Citation) Author Title | Country | Study Type | Main Findings |
| (8) Oluwole et al  Household biomass fuel use, asthma symptoms severity, and asthma underdiagnosis in rural schoolchildren in Nigeria: A cross-sectional observational study | Nigeria | Cross sectional observational community survey on schoolchildren | Underdiagnosis of asthma 2.2% diagnosed asthma vs 24.4% with asthma symptoms. Underdiagnosis due to inability to recognise symptoms by children and caregivers. In-accessibility to healthcare facilities |
| (18) Oluwole et al Asthma diagnosis among children along an urban-rural gradient. | Nigeria | Cross sectional among school children | Underdiagnosis of asthma along a rural urban gradient caused by inaccessibility to health facilities.. |
| (19) Zar , Levin . Challenges in Treating Pediatric Asthma in Developing Countries | South Africa | Review | The terms ‘asthma’ and ‘wheeze’ are absent in some local, ethnic languages. Poor socio-economic status and/or lack of medical health funding, diagnostic tests, was associated with under-recognition of asthma hence severe asthma. |
| (20) Masekela et al. The increasing burden of asthma in South African children: A call to action. | South Africa | Review | Up to 50% of children in urban communities have severe asthma symptoms, and many asthmatics lack a formal diagnosis and thus access to treatment. Severe wheezing in children due to exposure to tobacco smoke. |
| (22) Nantanda et al Asthma and Pneumonia among Children Less Than Five Years with Acute Respiratory Symptoms in Mulago Hospital, Uganda: Evidence of Under-Diagnosis of Asthma. | Uganda | Cross sectional study of children under 5years of age attending emergency department | Children with ‘asthma syndrome’ misdiagnosed as pneumonia |
| (33) Amorha et al Knowledge, attitudes and quality of life of caregivers towards asthma in their children: a Nigerian perspective. | Nigeria | Cross sectional study on caregivers of hospitalized asthmatic children | Only 27.5% of caregivers knew that cough , fastbreathing and wheeze are the main symptoms of asthma |
| (34) van Gemert F, Chavannes N, Nabadda N, Luzige S, Kirenga B, Eggermont C, et al. Impact of chronic respiratory symptoms in a rural area of sub-Saharan Africa: an in-depth qualitative study in the Masindi district of Uganda. | Uganda | Qualitative study. Focus group discussion on beliefs, and attitudes of varying community clusters towards chronic respiratory symptoms | Underdiagnosis of asthma caused by stigma associated with the presence of respiratory symptoms leading to caregivers not reporting symptoms |
| (35) Ayuk et al Spirometry in Asthma Care: A Review of the Trends and Challenges in Pediatric Practice. | Nigeria | Systematic review of literature in SSA | Unavailability of spirometers. Ignorance of their importance in diagnosis and follow-up by health workers. |
| (36) Ozoh et al Knowledge and practice assessment, and self reported barriers to guideline based asthma management among doctors in Nigeria | Nigeria | Cross sectional study among doctors | Only 42% of doctors had good level of asthma knowledge. |
| (37) Chima et al A survey of asthma management practices and implementation of Global Initiative for Asthma guidelines among doctors in a resource-limited setting in Nigeria | Nigeria | Cross sectional among hospital doctors | 23.9% of doctors had used of spirometry |
| (38) Desalu et al Asthma in Nigeria: Are the facilities and resources available to support internationally endorsed standards of care? | Nigeria | Cross sectional Survey among physicians working in 68 tertiary hospitals | Most tertiary hospital do not have respiratory physicians, paediatricians, asthma clinics, asthma registers, asthma protocols. Only 38% had peak flow meter, 29.4 had spirometer, 15.6% had skin allergy test kits. |
| (39) Desalu et al Evaluation of Current Knowledge, Awareness and Practice of Spirometry among Hospital -based Nigerian Doctors. | Nigeria | Cross Sectional study among hospital doctors | Lack of awareness of usefulness of spirometry in asthma diagnosis. |
| (40) Kibirige D, Kampire L, Atuhe D, Mwebaze R, Katagira W, Muttamba W et. Access to affordable medicine and diagnostic test for asthma and COPD in sub Saharan Africa: The Ugandan perspective. | Uganda | This was a cross sectional study in which the availability of diagnostic tests was assessed | Recommended that there should be strategies to improve access to asthma diagnostic tests. |
| (41) Kibirige et al Availability and affordability of medicines and diagnostic tests recommended for management of asthma and chronic obstructive pulmonary disease in sub-Saharan Africa: a systematic review. | Uganda (SSA) | Systematic Review | Lack of diagnostic equipment. Availability of spirometry ranging from 13% to 53% in most countries in Sub Saharan Africa. |
| (42) Nwosu et al Current pattern of spirometry utilisation in a sub-Saharan African country. | Nigeria | Retrospective study of patients who underwent spirometry over two and half years between January 2013 and June 2015 in the University of Nigeria Teaching Hospital (UNTH). | Low referral of patients from primary care health workers for spirometry. |
| (43) Obaseki et al Gaps in Capacity for Respiratory Care in Developing Countries. Nigeria as a Case Study | Nigeria | Cross sectional study, pediatric and adult respiratory physicians | Lack of respiratory physicians.  Lack of diagnostic equipment |
| (44) Osaretin et al Asthma management by medical practitioners: the situation in a developing country | Nigeria | Cross sectional study among medical practitioners | Up to 88.5% of medical practitioners practiced inappropriate asthma diagnosis. |
| (45) Ayuk et al  Use of Global initiative for asthma (GINA) guidelines in asthma management among paediatric residents in a Sub Saharan African country: a cross-sectional descriptive study. | Nigeria | Cross sectional study among paediatric residents | Non adherence to GINA guidelines |
| (46) Nantanda R, Ostergaard MS, Ndeezi G, Tumwine JK. Factors associated with asthma among under-fives in Mulago hospital, Kampala Uganda: a cross sectional study. | Uganda | Cross sectional study among under 5 children with respiratory symptoms | Overdiagnosis of pneumonia and bronchiolitis over asthma |
| **Objective 2: Solutions to challenges in asthma diagnosis (N=16)** | | | |
| (Citation) Author Title | Country | Study Type | Findings |
| (8) Oluwole et al Household biomass fuel use, asthma symptoms severity, and asthma underdiagnosis in rural schoolchildren in Nigeria: a cross-sectional observational study. | Nigeria | Cross sectional, observational | Increase awareness of asthma symptoms by educating communities |
| (20) Masekela et al. The increasing burden of asthma in South African children: A call to action | South Africa | Review of literature | Community education to increase knowledge among parents and children |
| (22) Nantanda et al Asthma and Pneumonia among Children Less Than Five Years with Acute Respiratory Symptoms in Mulago Hospital, Uganda: Evidence of Under-Diagnosis of Asthma. | Uganda | Cross sectional study of children under 5years of age attending emergency department | Need for diagnostic tests that differentiate asthma from pneumonia |
| (34) van Gemert F, Chavannes N, Nabadda N, Luzige S, Kirenga B, Eggermont C, et al. Impact of chronic respiratory symptoms in a rural area of sub-Saharan Africa: an in-depth qualitative study in the Masindi district of Uganda. | Uganda | Qualitative study. Focus group discussion on beliefs, and attitudes of varying community clusters towards chronic respiratory symptoms | Need for community based education programmes to destigmatise chronic respiratory conditions including asthma. |
| (35) Ayuk et al Spirometry in Asthma Care: A Review of the Trends and Challenges in Pediatric Practice. | Nigeria | Systematic review of literature in SSA | Need to improve availability of spirometry and education of health workers on importance of spirometry in diagnosis and follow-up. |
| (38) Desalu et al Asthma in Nigeria: Are the facilities and resources available to support internationally endorsed standards of care? | Nigeria | Cross sectional Survey among physicians working in 68 tertiary hospitals | Availing spirometry to confirm asthma diagnosis. Training health workers on use of spirometry. |
| (42) Nwosu et al Current pattern of spirometry utilisation in a sub-Saharan African country. | Nigeria | Retrospective study of patients who underwent spirometry over two and half years between January 2013 and June 2015 in the University of Nigeria Teaching Hospital (UNTH). | Education on primary health care workers on referral of patients from primary care health workers for spirometry. |
| (43) Obaseki et al Gaps in Capacity for Respiratory Care in Developing Countries. Nigeria as a Case Study | Nigeria | Cross sectional study, pediatric and adult respiratory physicians | Need for skills training including training of respiratory physicians. |
| (47) Temitayo I.O, Adegbenro, Caleb A ,Awopeju, Olayemi F , Olatona, Foluke A. Knowledge and Perceptions of Asthma in a Nigerian High School. | Nigeria | Cross- sectional study which utilized a pre-tested 71- item, self- administered questionnaires to collect information on knowledge and perceptions of the respondents on asthma | Education programmes for students on asthma.  Recommendation to develop a school based asthma education curriculum. |
| (48) Kuti B, Kuti D, Omole K, Mohammed L, Ologun B, Oso B. Prevalence and factors associated with exercise-induced bronchospasm among rural school children in Ilesa, Nigeria. | Nigeria | In a cross sectional study to determine the prevalence of exercise induced none of the 9.2% had previously been diagnosed with asthma. | Recommended routine screening of these children for early diagnosis |
| (49) Østergaard et al Childhood asthma in low income countries: an invisible killer? | Uganda | Review of literature | Redefinition of WHO definition of pneumonia to include fever  Need for research on low cost management strategies for children with respiratory diseases |
| (50) du Plessis, Gerber and Brand . Managing asthma in primary care through imperative outcomes | South Africa | Retrospective review of patient records | Need for education of health workers on guideline based asthma management |
| (51) van Niekerk A, White DA, Goussard P, Risenga SM. Childhood asthma: A best-practice strategy for diagnosis and assessment of control in South Africa. | South Africa | Review of evidence | Proposed a four-step diagnostic approach to asthma in preschool and school age children. |
| (52) Nantanda et al Symptom-based screening tool for asthma syndrome among young children in Uganda | Uganda | Cross sectional study of children under 5 attending emergency department | Symptom based questionnaires may serve as alternative to asthma diagnosis in resource poor settings.  Need to test validity and reliability |
| (53) Murdoch J, Curran R, Cornick R, Picken S, Bachmann M, Bateman E, et al. Addressing the quality and scope of paediatric primary care in South Africa: evaluating contextual impacts of the introduction of the Practical Approach to Care Kit for children (PACK Child) | South Africa | Qualitative survey of the uptake of the Pack child | Proposed need for Primary health care to adopt PACK child which include algorithms for asthma diagnosis and training of health workers |
| (54) Adeyeye et al Five-year audit of spirometry at the LASUTH, Ikeja, south-west Nigeria | Nigeria | Retrospective study on utilization of spirometry services at Lagos State University teaching hospital | Increase awareness on use of spirometry |
| (55) Masekela et al. The diagnosis of asthma in children: An evidence-based approach to a common clinical dilemma. | South Africa | Review of literature | Need for algorithms for asthma diagnosis based on exclusion of all other causes of wheeze, trial of inhaled steroids especially where confirmatory lung function tests cannot be done |
