# Supplementary material for: Challenges in the diagnosis of asthma in children, what are the solutions? A scoping review of 3 countries in sub Saharan Africa
Source: Respir Res. 2022 Sep 19;23:254. doi: 10.1186/s12931-022-02170-y (PMC9487077; doi:10.1186/s12931-022-02170-y)
Supplement: Supplementary file 3 — Additional file 3. PRISMA 2020 flow diagram for new systematic reviews which included searches of databases and registers only. [file 12931_2022_2170_MOESM3_ESM.docx]

**Identification of studies via databases**

Records removed *before screening*:

Duplicate records removed (n =64 )

Articles identified from*:

Database Search

Search 1 (n=168)

Search 2 (n=122)

**Identification**

Articles assessed for eligibility by screening titles

n=226

Articles excluded**

(n = 104)

Abstracts assessed for eligibility

(n = 122)

Articles not retrieved

(n =29 )

**Screening**

Full articles analysed against eligibility criteria

(n = 93 )

Articles excluded:

Reason 1 (n =32) not done in 2010 to 2021 )

Reason 2 (n =20) not from Nigeria, South Africa or Uganda )

Reason 3 (n =16) did not meet the eligibility criteria )

etc.

Studies included in review

n=28

**Included**

*Consider, if feasible to do so, reporting the number of records identified from each database or register searched (rather than the total number across all databases/registers).

**If automation tools were used, indicate how many records were excluded by a human and how many were excluded by automation tools.

*From:*  Page MJ, McKenzie JE, Bossuyt PM, Boutron I, Hoffmann TC, Mulrow CD, et al. The PRISMA 2020 statement: an updated guideline for reporting systematic reviews. BMJ 2021;372:n71. doi: 10.1136/bmj.n71

For more information, visit: <http://www.prisma-statement.org/>
